# The efficacy and safety of novel antiepileptic drugs in treatment of epilepsy of patients with brain tumors

**DOI:** 10.3389/fneur.2024.1344775

**Published:** 2024-03-07

**Authors:** Weiwei Zhai, Qiaoling Yu, Huizhen Wu

**Affiliations:** ^1^Graduate School of Hebei Medical University, Shijiazhuang, China; ^2^National Clinical Drug Monitoring Center, Department of Pharmacy, Hebei General Hospital, Shijiazhuang, China

**Keywords:** antiepileptic drugs, BTRE, perampanel, lacosamide, single-arm meta-analysis

## Abstract

**Objective:**

This meta-analysis aimed to assess the effectiveness and safety of novel antiepileptic drugs (AEDs) in treating epilepsy in patients with brain tumors (BTRE).

**Methods:**

A search was conducted on PubMed, EMBASE, Web of Science, and the Cochrane Library from inception to February 2023, with English language restriction.

**Results:**

In this meta-analysis, 18 clinical trials involving 755 BTRE patients were included to assess the efficacy and safety of novel AEDs in BTRE treatment. At the last follow-up, a ≥50% reduction in seizure frequency was experienced by 72% of patients (random-effects model, 95% CI = 0.64–0.78) using novel AEDs. At the last follow-up, seizure freedom was experienced by 34% of patients (random-effects model, 95% CI = 0.28–0.41) using novel AEDs. The pooled incidence of AEs was found to be 19% (95% CI: 13%–26%), with a withdrawal rate due to adverse effects of only 3%. Comparable efficacy and incidence of adverse effects were observed between lacosamide and perampanel.

**Conclusion:**

This meta-analysis suggests that novel antiepileptic drugs are deemed effective for seizure control in brain tumor patients, particularly when used as adjunctive therapy. Although lacosamide and perampanel received more focus in studies, no significant difference was observed in the efficacy and adverse reactions of these two drugs in seizure control. Further randomized controlled trials are deemed necessary to validate our findings.

## 1 Introduction

Brain and central nervous system (CNS) tumors rank among the most fatal cancers, causing significant morbidity and mortality in the United States (1). Epilepsy emerges as the predominant symptom in individuals with brain tumors, and a single seizure is sufficient for diagnosing epilepsy in this context (2). The likelihood of brain tumor-related epilepsy (BTRE) is markedly influenced by tumor histology (3). Over 80% of diffuse low-grade glioma patients experience BTRE, while glioblastoma and meningioma patients exhibit seizure rates of 62%–68% and 40%–47%, respectively (4–6). Seizures in brain tumor patients stem from various factors, including mechanical compression of tumors, disturbances in tumor vascularization and oxygen demand, inflammatory processes, and neurotransmitter imbalances (3). Even after complete tumor removal, eradicating epilepsy proves challenging, as seizures in BTRE are partially generated by surrounding tissues. Antiepileptic drugs (AEDs), possessing known pharmacological properties, the ability to traverse the blood-brain barrier, and established efficacy in seizure control, also demonstrate potential anti-tumor activity (7). Consequently, AEDs, with proven efficacy and safety and no heightened cancer risk, are identified as optimal for BTRE treatment (8). AED selection in BTRE adheres to principles such as potential beneficial side effects, availability of intravenous formulations with rapid onset, avoidance of adverse effects, and minimizing drug interactions (3). Thus, the choice of current AEDs in BTRE should prioritize considerations of pharmacokinetics, pharmacodynamics, tolerability, and side effects.

Novel AEDs, encompassing lacoxamide, perampanel, brivaracetam, rufinamide, cannabidiol, cenobanmate, retigabine, eslicarbazepine acetate, and stiripentol, exhibit notable efficacy in epilepsy control and treatment. However, the debate persists regarding their efficacy and safety in the context of brain tumor-related epilepsy. Consequently, this study aims to assess the effectiveness and safety of novel AEDs in treating epilepsy in patients with brain tumors.

## 2 Methods

### 2.1 Search strategy

We conducted a literature search in four databases, namely Embase, PubMed, Web of Science, and the Cochrane Library, from their inception to February 2023, with a restriction to English-language publications. The keywords employed were “perampanel,” “rufinamide,” “cannabidiol,” “cenobanmate,” “retigabine,” “eslicarbazepine acetate,” “brivaracetam,” “stiripentol,” “lacosamide,” and “brain neoplasms.”

### 2.2 Study selection

Inclusion criteria were as follows: (1) study type: randomized controlled trial (RCT), prospective clinical trials, retrospective cohort studies, prospective case–control studies, and case series (≥5); (2) diagnosis: patients diagnosed with brain tumor-related epilepsy (BTRE); (3) drug: perampanel, rufinamide, retigabine, eslicarbazepine acetate, stiripentol, lacosamide, brivaracetam, regardless of dosage, administration method, and duration; and (4) data: information on the reduction of seizure frequency, including seizure reduction ≥50% from baseline, seizure freedom, and adverse events (AEs).

Exclusion criteria were as follows: (1) *in vitro* or animal experiments; (2) inability to extract exact data from the article; (3) conference abstracts; (4) number of participants <5.

### 2.3 Data extraction

Two authors independently screened titles and abstracts of relevant articles based on established inclusion and exclusion criteria, thoroughly reviewing literature of high relevance. Subsequently, data were extracted from articles meeting the research criteria.

Information extracted from the included studies included: (1) study information: first author, publication year, and study design; (2) patient information: number of participants, gender, and age; (3) treatment information: drug, route of administration, dose, duration, and combination of treatment; (4) primary outcomes: seizure reduction ≥50% from baseline, seizure freedom, and AEs.

### 2.4 Quality assessment

For included non-randomized controlled trials, we utilized the Methodological Index for Non-Randomized Studies (MINORS) to assess quality (9).

### 2.5 Statistical analysis

Heterogeneity was evaluated using the Cochran *Q* test and *I*^2^ statistics. Different effect models were selected based on *I*^2^ statistics, utilizing the random effect model when *I*^2^ exceeded 50%, and the fixed effect model otherwise. Sensitivity analysis and subgroup analyses were conducted to explore the source of heterogeneity. Publication bias analysis employed funnel plots and Egger’s test. Two-proportion *z*-tests were used to compare pooled proportions. All analyses were conducted using R (Version 4.3.1), with a significance level set at *p* < 0.05.

## 3 Results

### 3.1 Studies selection and characteristics

After an initial search, we identified a total of 1,021 articles across four databases: 835 from Embase, 33 from PubMed, 146 from Web of Science, and 7 from the Cochrane library. The first step involved removing duplicate records obtained from the database searches. In the second step, we conducted a preliminary screening by reviewing the titles and abstracts of the retrieved articles. The third step involved the final identification of articles meeting the inclusion criteria through a thorough review of their full texts. After excluding 122 duplicate records, two independent researchers screened the titles and abstracts of the remaining 899 articles and excluded 252 studies that were unrelated to our objectives.

**Figure 1:**
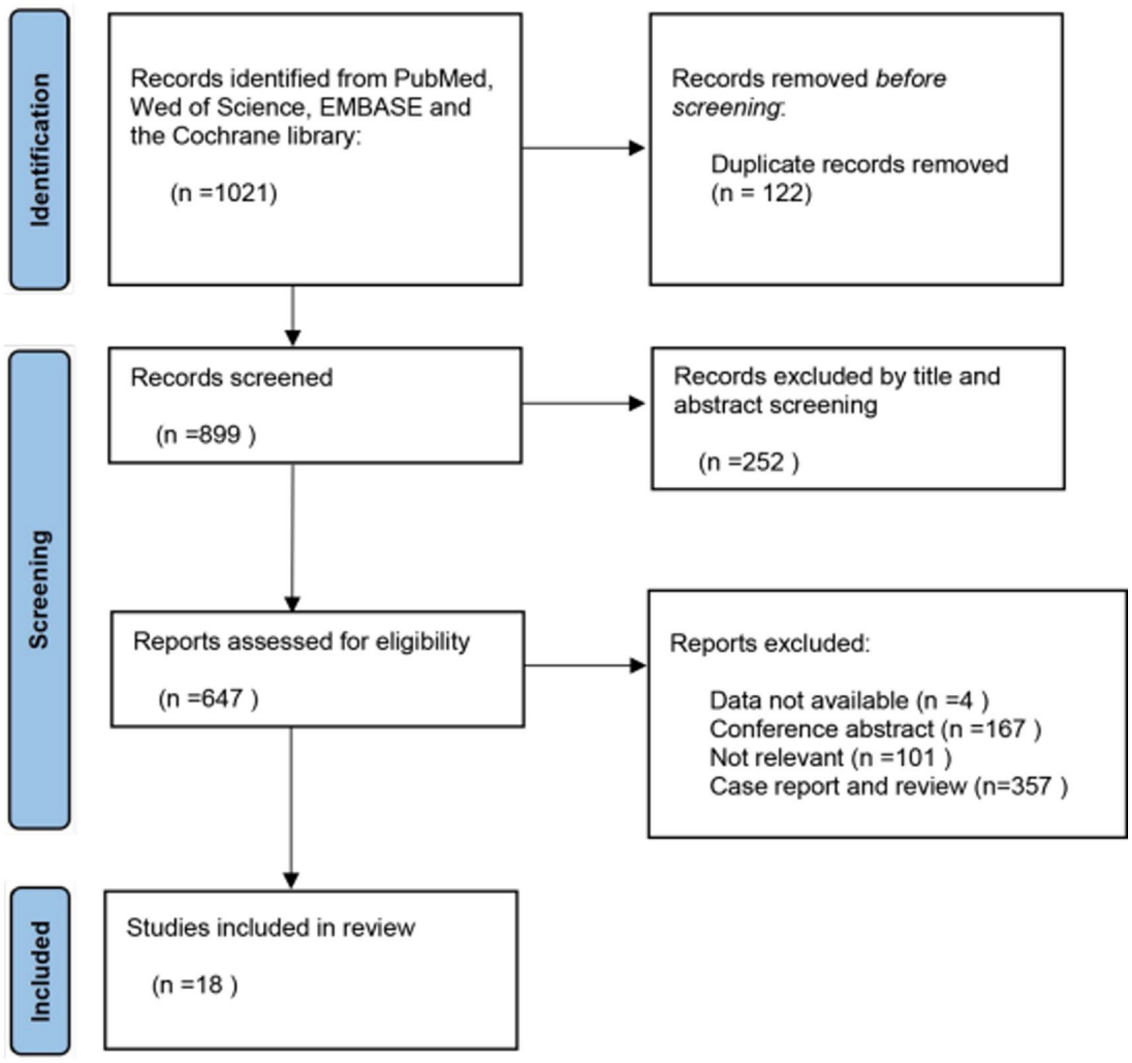
Flow diagram of study selection.

Upon reading the full text of the remaining 647 articles, 629 were excluded, resulting in a total of 18 articles included in our meta-analysis (10–27). The PRISMA flow chart illustrating the study selection process is presented in Figure 1.

Nine studies employed lacosamide, six studies utilized perampanel, and one each utilized eslicarbazepine, brivaracetam, and clobazam. The publication years ranged from 2011 to 2022. Detailed baseline characteristics of the included clinical trials in this meta-analysis are presented in Table 1.

**Table 1:** Baseline characteristics of included studies.

Author	Year	No. (male)	Type of tumor	Seizure type	Drug	Mean dose	Concomitant AEDs (%)	Follow-up period (month)	NOS
Ruda et al.	2020	93(47)	Primary brain tumor	BTRE	Lacosamide	229.0 mg/day	100%	6	6
Saria et al.	2013	70(40)	Primary brain tumor	BTRE	Lacosamide	274.9 mg/day	87.1%	6.2	6
Sepulveda et al.	2017	39 (26)	Primary and metastases brain tumor	BTRE	Lacosamide	138.5 mg/day	89.7%	3/6	7
Maschio et al.	2019	11	Primary brain tumor	BTRE	Perampanel	7.3 mg/day	100%	12	5
Toledo et al.	2018	48 (34)	Primary and metastases brain tumor	BTRE	Lacosamide	200 mg/day	100%	30.5	7
Maschio et al.	2011	14 (8)	Primary brain tumor	Uncontrolled seizures	Lacosamide	332.1 mg/day	100%	5.4	6
Ruda et al.	2018	71 (48)	Primary brain tumor	Uncontrolled seizures	Lacosamide	200 mg/day	100%	3/6/9	6
Zoccarato et al.	2021	8 (5)	Primary brain tumor	BTRE	Eslicarbazepine acetate	950 mg/day	100%	3/6/9	5
Maschio et al.	2020	33 (19)	Primary brain tumor	BTRE	Brivaracetam	175 mg/day	100%	10	5
Heugenhauser et al.	2021	5 (3)	Primary brain tumor	Uncontrolled seizures	Perampanel	6.8 mg/day	100%	30.2	5
Coppola et al.	2020	36 (13)	Primary brain tumor	Uncontrolled seizures	Perampanel	6.5 mg/day	100%	12	5
Izumoto et al.	2018	12 (8)	Primary brain tumor	Uncontrolled seizures	Perampanel	5 mg/day	100%	6	5
Maschio et al.	2017	25 (18)	Primary brain tumor	Uncontrolled seizures	Lacosamide	300 mg/day	100%	6	5
Mo et al.	2022	132 (76)	Primary brain tumor	BTRE	Lacosamide	250 mg/day	62.9%	3/6	6
Villanueva et al.	2016	105 (65)	Primary and metastases brain tumor	BTRE	Lacosamide	350 mg/day	97.1%	3/6	7
Vecht et al.	2017	12 (9)	Primary brain tumor	Uncontrolled seizures	Perampanel	8 mg/day	91.7%	6	6
Brahmbhatt et al.	2021	35 (26)	Primary brain tumor	BTRE	Clobazam	20.4 mg/day	100%	6/12/24	6
Maschio et al.	2020	24 (16)	Primary and metastases brain tumor	Uncontrolled seizures	Perampanel	6.6 mg/day	100%	6	5

### 3.2 Seizure reduction

**Figure 2:**
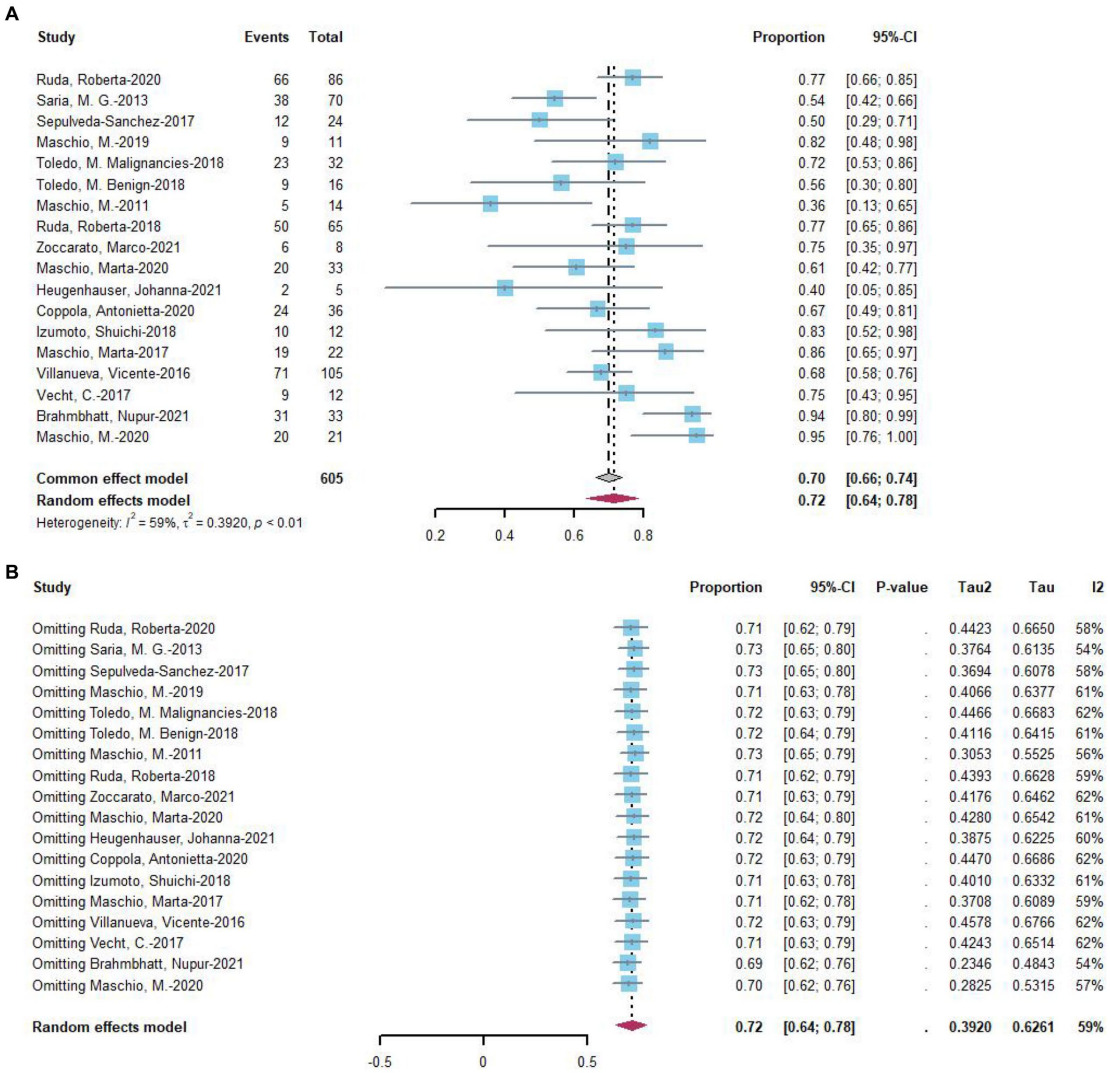
**(A)** Forest plot of last follow-up seizure reduction ≥50% with the most data. **(B)** The sensitive analysis of last follow-up seizure reduction ≥50% with the most data.

A treatment response was defined as a reduction of more than 50% in seizures, and this outcome was assessed in 17 studies. At the last follow-up with the most data, 72% (random-effects model, 95% CI = 0.64–0.78) of 605 patients undergoing novel AEDs experienced a treatment response in seizure frequency (Figure 2A). A linear regression test assessing funnel plot asymmetry for the 17 studies revealed no significant publication bias (*p* = 0.28). Following a sensitive analysis (Figure 2B), one study was excluded, and the adjusted pooled estimate of data was 0.69 (95% CI: 0.64–0.78).

Subgroup analysis was conducted on 17 studies that exhibited a response in seizures. The analysis suggested that the choice of drug might be a significant source of heterogeneity (Table 2). Following subgroup analysis, brain tumor type (*p* = 0.9) and epilepsy type (*p* = 0.54) were not identified as sources of heterogeneity. The response rate in seizures was 0.66 with lacosamide (*n* = 7) and 0.78 with perampanel (*n* = 6). The effective rate of eslicarbazepine acetate for a 50% reduction in seizures was 0.75, that of brivaracetam was 0.61, and that of clobazam was 0.94. However, there was only one study for each of these three drugs, with limited data. Additionally, four studies included patients on monotherapy, prompting subgroup analyses with or without concomitant medication. Subgroup analysis indicated that concomitant medications or lack thereof might be a significant source of heterogeneity (Table 2). The response rate in the combination arm was 75%, while the response rate in the uncoadministered group was 61%.

**Table 2:** Subgroup analysis of pooled estimates of seizure response.

Subgroup	No. of studies	Mean	95% CI	*p*-value between subgroups
**Drug**				
Lacosamide	7	66%	0.57–0.74	0.04
Perampanel	6	78%	0.64–0.87	
Eslicarbazepine acetate	1	75%	0.35–0.97	
Brivaracetam	1	61%	0.42–0.77	
Clobazam	1	94%	0.80–0.99	
**Brain tumor type**				
Primary brain tumor	14	71%	0.62–0.79	0.9
Primary and metastases brain tumor	4	73%	0.54–0.86	
**Epilepsy type**				
BTRE	9	69%	0.60–0.77	0.54
Uncontrolled seizures	8	74%	0.60–0.85	
**Concomitant or not**				
Concomitant	13	75%	0.65–0.82	0.03
Non-concomitant	4	61%	0.64–0.78	

**Figure 3:**
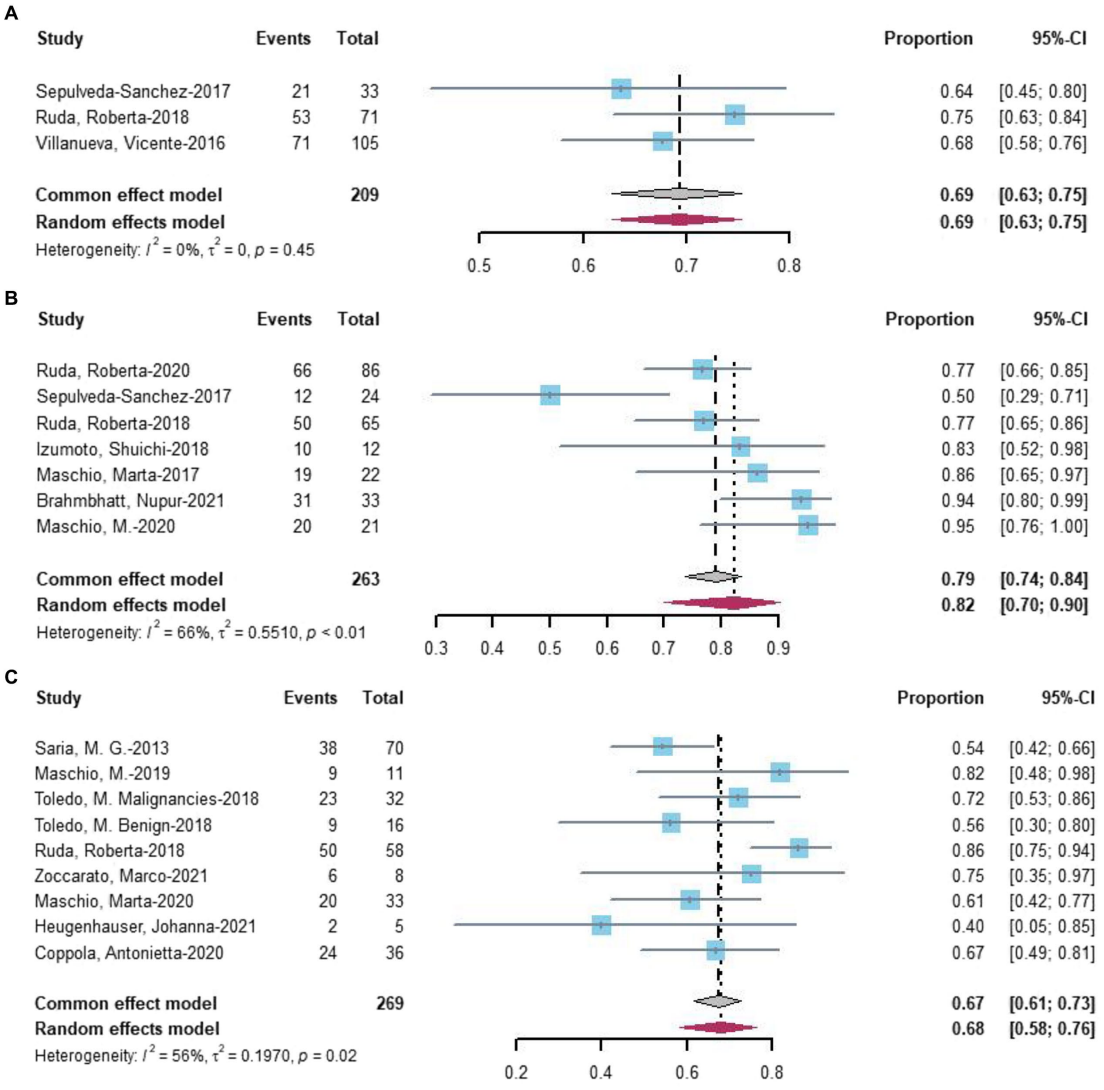
Forest plot showing the **(A)** 3 months, **(B)** 6 months, and **(C)** more than 6 months seizure reduction ≥50%.

Three studies provided data on treatment response in seizures at 3 months of follow-up, with 69% of 209 patients experiencing a treatment response (Figure 3A). For 6 months of follow-up, data from seven studies revealed that 82% of 263 patients had a treatment response (Figure 3B). At more than 6 months of follow-up, eight studies reported that 68% of 269 patients had a treatment response (Figure 3C). Two-proportion z-tests indicated a significant difference in treatment response between 3 months and 6 months of follow-up (69.4% vs. 78.1%, *p* < 0.05). Additionally, a significant difference was observed in treatment response between 6 months and more than 6 months of follow-up (79.1% vs. 67.3%, *p* < 0.05), but no significant difference was found between 3 months and more than 6 months of follow-up (*p* = 0.70).

**Figure 4:**
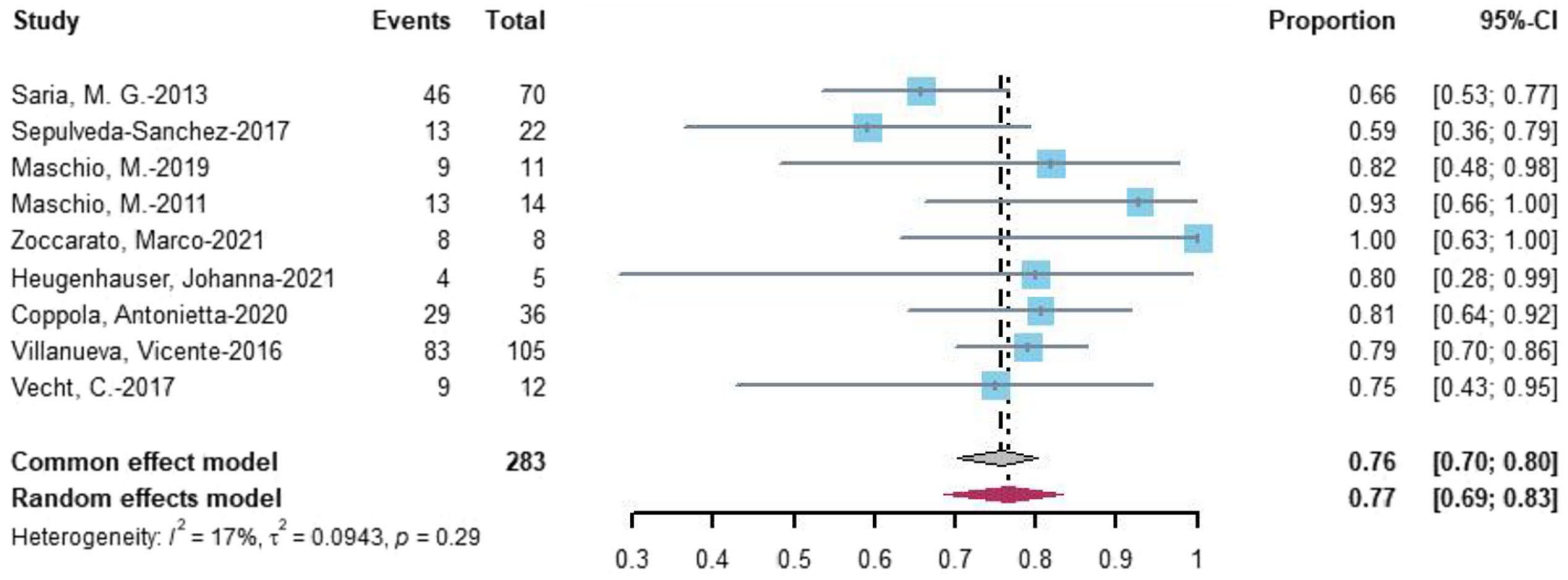
Forest plot of last follow-up seizure reduction with the most data.

Nine studies evaluated seizure reduction outcomes. At the last follow-up with the most data, 77% (random-effects model, 95% CI = 0.69–0.83) of 283 patients undergoing novel AEDs experienced seizure reduction (Figure 4).

### 3.3 Seizure freedom

**Figure 5:**
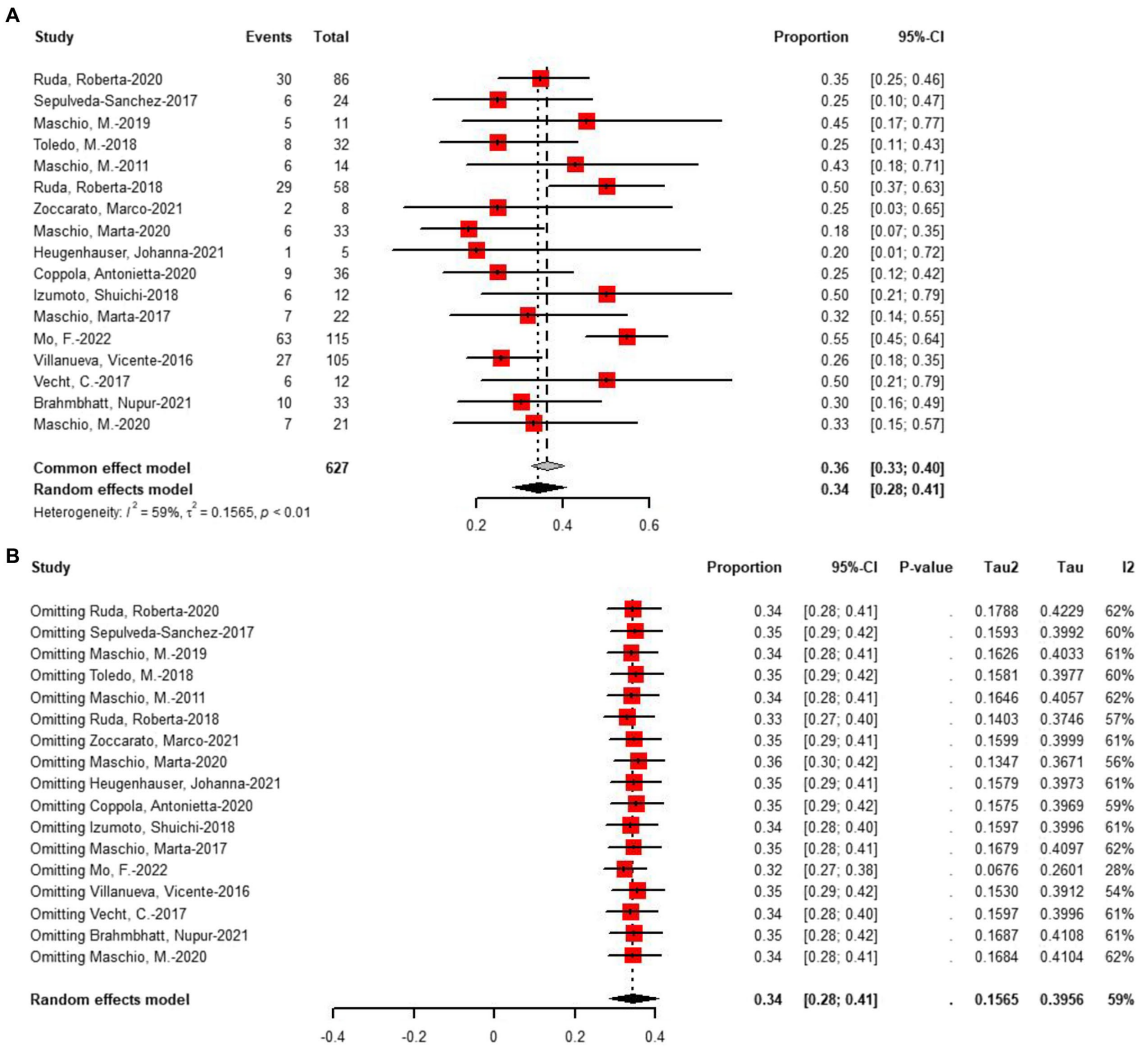
**(A)** Forest plot of last follow-up seizure freedom with the most data. **(B)** The sensitive analysis of last follow-up seizure freedom with the most data.

Seventeen studies assessed seizure freedom. At the last follow-up with the most data, 34% (random-effects model, 95% CI = 0.28–0.41) of 627 patients undergoing novel AEDs experienced seizure freedom (Figure 5A). A linear regression test assessing funnel plot asymmetry for the 17 studies revealed no significant publication bias (*p* = 0.20). After a sensitive analysis (Figure 5B), one study was excluded, and the adjusted pooled estimate of data was 0.32 (95% CI: 0.27–0.38).

Subgroup analysis was conducted on 17 studies that reported seizures freedom. The analysis suggested that the type of tumor studied might be a significant source of heterogeneity (Table 3). After subgroup analysis, drug (*p* = 0.37) and epilepsy type (*p* = 0.25) were not identified as sources of heterogeneity. The rate of seizure freedom was 0.37 with primary brain tumor (*n* = 13) and 0.26 with primary and metastatic brain tumor (*n* = 4). Additionally, four studies included patients on monotherapy, prompting subgroup analyses with or without concomitant medication. Subgroup analysis indicated that concomitant medications or lack thereof may not be a significant source of heterogeneity (*p* = 0.58).

**Table 3:** Subgroup analysis of pooled estimates of seizure freedom.

Subgroup	No. of studies	Mean	95% CI	*p*-value between subgroups
**Drug**				
Lacosamide	8	36%	0.28–0.46	0.37
Perampanel	6	35%	0.26–0.45	
Eslicarbazepine acetate	1	25%	0.03–0.66	
Brivaracetam	1	18%	0.07–0.35	
Clobazam	1	30%	0.16–0.49	
**Brain tumor type**				
Primary brain tumor	13	37%	0.30–0.45	0.03
Primary and metastases brain tumor	4	26%	0.20–0.33	
**Epilepsy type**				
BTRE	9	32%	0.24–0.41	0.25
Uncontrolled seizures	8	39%	0.30–0.48	
**Concomitant or not**				
Concomitant	13	33%	0.27–0.40	0.58
Non-concomitant	4	38%	0.24–0.53	

**Figure 6:**
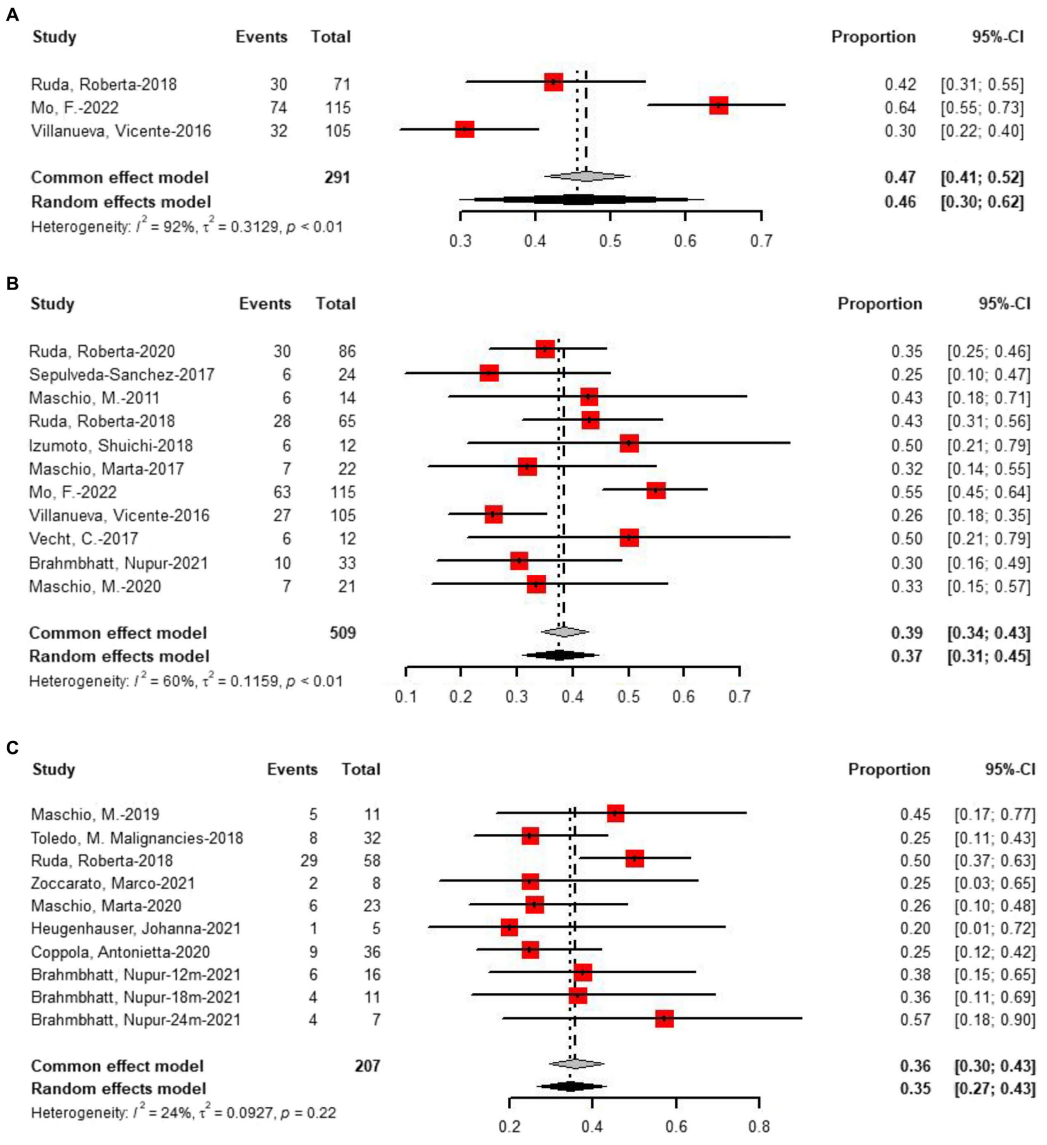
Forest plot showing the **(A)** 3 months, **(B)** 6 months, and **(C)** more than 6 months seizure freedom.

Three studies provided data on treatment seizures freedom at 3 months of follow-up, with 46% of 291 patients achieving seizures freedom (Figure 6A). For 6 months of follow-up, data from 11 studies revealed that 37% of 509 patients had seizures freedom (Figure 6B). At more than 6 months of follow-up, eight studies reported that 35% of 207 patients had seizures freedom (Figure 6C). Two-proportion *z*-tests indicated a significant difference in treatment response between 3 months and 6 months of follow-up (46% vs. 37%, *p* < 0.01). Additionally, a significant difference was observed in treatment response between 6 months and more than 6 months of follow-up (37% vs. 35%, *p* < 0.05), but no significant difference was found between 3 months and more than 6 months of follow-up (*p* = 0.55).

### 3.4 Treatment-related adverse events

**Figure 7:**
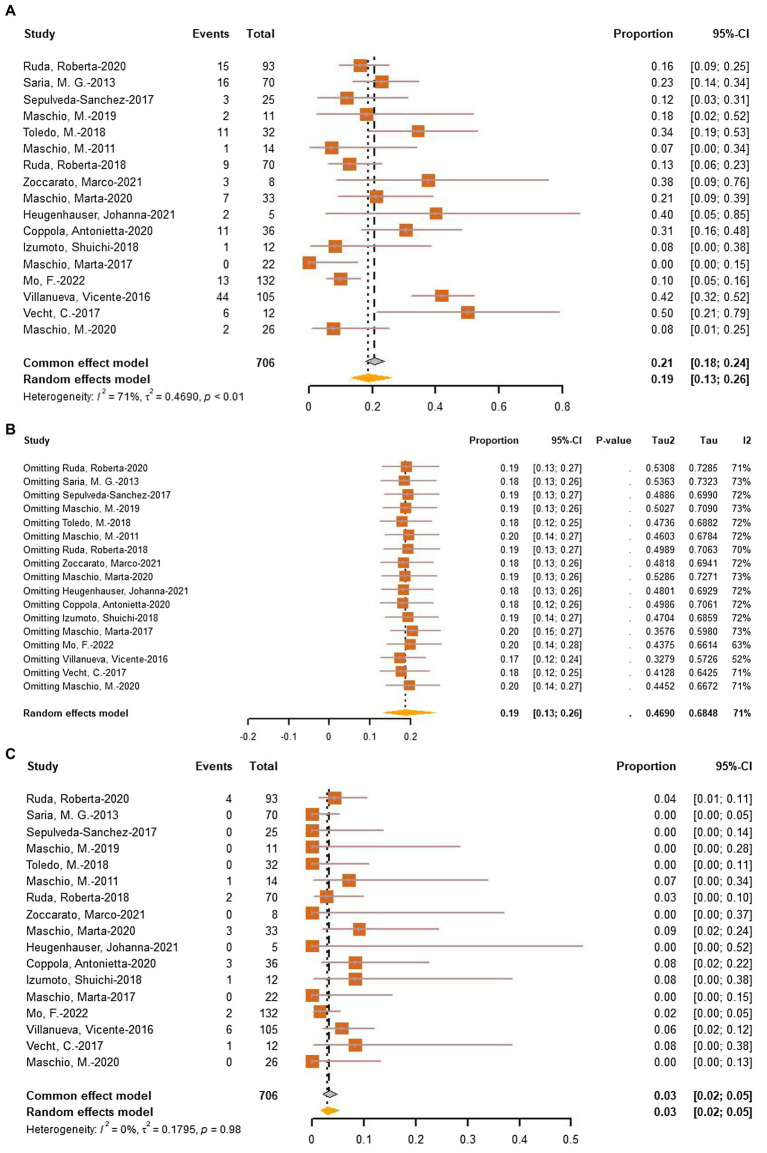
**(A)** Forest plot of last follow-up adverse effects with the most data. **(B)** The sensitive analysis of last follow-up adverse effects. **(C)** Forest plot of last follow-up withdrawal due to adverse effects.

To assess the incidence of AEDs-related adverse events (AEs), a meta-analysis of AEs (706 patients in 17 studies) and withdrawals due to adverse effects (706 patients in 17 studies) was performed. The pooled incidence of AEs was 19% (95% CI: 13–26%, *I*^2^ = 71%, *p* < 0.01, Figure 7A). Following a sensitive analysis (Figure 7B), one study was excluded, and the adjusted pooled estimate of data was 0.17 (95% CI: 0.12–0.24). After subgroup analysis, drug (*p* = 0.57), type of tumor (*p* = 0.54), and epilepsy type (*p* = 0.31) were not identified as sources of heterogeneity. The rate of withdrawal due to adverse effects was only 3% (95% CI: 2%–5%, *I*^2^ = 0%, *p* < 0.01, Figure 7C).

### 3.5 Lacosamide vs. perampanel

At the last follow-up, the seizure response rate of perampanel was 78% (95% CI = 0.64–0.87), and the seizure response rate of lacosamide was 66% (95% CI = 0.57–0.74). Two-proportion *z*-tests revealed no significant difference in response rates between perampanel and lacosamide (*p* = 0.12). Regarding seizure freedom, at the last follow-up, the seizure freedom rate of perampanel was 35% (95% CI = 0.26–0.45), and the seizure freedom rate of lacosamide was 36% (95% CI = 0.28–0.46). Two-proportion z-tests revealed no significant difference in seizure freedom rates between perampanel and lacosamide (*p* = 0.59). While other drugs may show significant differences, they were less studied. Concerning the incidence of adverse effects, the incidence of adverse effects for perampanel was 21%, and for lacosamide, it was 17%. However, two-proportion z-tests revealed no significant difference in adverse effects between perampanel and lacosamide (*p* = 0.84).

## 4 Discussion

BTRE is prevalent among patients with brain tumors, yet there is no universally agreed-upon AED treatment for BTRE. Levetiracetam, lamotrigine, and topiramate have all been suggested as potential first-line AEDs for BTRE treatment (28). Studies indicate that levetiracetam serves as a suitable first-line AED for grade 2 glioma seizures compared to commonly used ASM such as carbamazepine, lamotrigine, sodium valproate, topiramate, and phenytoin. More than 60% of patients on levetiracetam achieve seizure freedom within 2 years, though it can lead to neuropsychiatric adverse reactions (29). BTRE significantly impacts patient morbidity and quality of life, yet its pathogenesis remains unclear. In high-grade gliomas, up to 15% to 25% of BTRE cases are resistant. Among various combinations, the dual administration of levetiracetam and valproic acid is deemed the most effective for refractory BTRE, despite more serious adverse reactions (30). Limited treatment options exist for brain tumors like high-grade gliomas. Standard medical therapy involves chemotherapy with temozolomide and radiation therapy. It is generally agreed upon that non-enzyme-inducing ASMs should be preferred to avoid interference with antineoplastic drugs and other medications like dexamethasone (31).

Children and adults with the most aggressive forms of brain cancer, malignant gliomas, or glioblastomas commonly develop brain edema, and this complication is usually treated with dexamethasone. Other research has shown that dexamethasone can reduce tumor microenvironment interference caused by, and even can inhibit glioma cell proliferation (32). Edema in patients with brain tumors may also lead to seizures, which can be controlled by dexamethasone by reducing edema. In patients with chemotherapy or dexamethasone, infection can persist throughout the disease, and infection can cause neurological decline or seizures (33). There is clear clinical evidence that dexamethasone can affect the pharmacokinetic parameters of phenytoin sodium (34). However, the commonly used AED in clinic is levetiracetam, and the literature supports that levetiracetam has no significant interaction with dexamethasone, acetaminophen and fentanyl (35). Changing the dose of dexamethasone may affect the control of seizures and thus the AED dose, and increasing the dose of dexamethasone may subsequently reduce the AED level. Similarly, care must be taken to monitor AED levels during dexamethasone tapering, as some patients develop AED toxicity following dexamethasone tapering. However, in the study of new AEDs, the influence of dexamethasone has not been deeply explored, and only one LAC study records and analyzes the dose change of dexamethasone (18).

The mechanism of action of the novel AEDs included in this study differs from that of traditional AEDs, making them less susceptible to metabolic enzyme interference. Consequently, these AEDs exhibit a low incidence of adverse reactions, most of which are tolerable. *In vitro* studies (36) suggest that PER possesses promising antitumor properties, although clinical data on its potential antitumor activity are lacking. Recent studies (37) report *in vitro* antitumor effects of lacosamide and brivaracetam on human glioma cells. More controlled trials and observational studies in larger cohorts are required to further explore the impact of these AEDs on tumor progression and comprehensively characterize their potential in BTRE patients. Among the novel AEDs, brivaracetam’s adverse effects occur less frequently (38). However, lacosamide, available in injectable form, holds an advantage in controlling acute episodes of BTRE.

In this meta-analysis, 18 clinical trials involving 755 patients with brain tumor-related epilepsy (BTRE) were incorporated to assess the efficacy and safety of novel AEDs in treating BTRE. Single-arm analysis revealed a treatment response rate of 72% and a seizure freedom rate of 34%. Moderate heterogeneity was observed in the response rate and seizure-free statistics across the included clinical studies, possibly attributed to variations in follow-up duration. All studies were either retrospective or prospective, utilizing subjective outcome indicators without blinding. Only five studies involved patients on single-drug administration, while others had previously undergone first-line antiepileptic therapy, shifting to novel AEDs due to adverse reactions or poor efficacy of other drugs. Sensitivity analysis indicated a significant difference in treatment response between monotherapy and combination therapy, suggesting better efficacy of novel AEDs as adjuvant therapy. However, no significant difference was observed in seizure freedom efficacy with or without combination therapy. The study by Brahmbhat et al. influenced the response rate statistics, likely due to the high response rate to clobazam treatment for BTRE. Exclusion of this study resulted in a decreased pooled response rate to 69% (95% CI: 0.62–0.76). In seizure-free statistics at the last follow-up, the study by Mo et al. was a source of consistency, likely due to the large number of patients on monotherapy. After excluding this study, the combined seizure-free rate decreased to 32% (95% CI: 0.27–0.38). The response rate and seizure-free rate at 6 months were 79.1% and 37%, respectively. The pooled incidence of adverse effects of novel AEDs for BTRE was 19%, with sensitivity analysis identifying heterogeneity as a potential source. Most adverse reactions in the included studies were dizziness, fatigue, and somnolence. Few patients withdrew due to adverse reactions. The higher rate of adverse effects in this study, particularly for LAC, compared to other studies, may be attributed to the higher dose. Despite more studies on LAC and PER, no significant difference in efficacy and adverse reactions between these two drugs in seizure control was observed.

Caution is advised in interpreting the results of our analysis. All studies included in this meta-analysis were either retrospective or prospective, introducing potential biases, particularly in retrospective trials. Well-designed randomized controlled trials are essential to validate our findings. Subgroup analysis based on tumor grade was not conducted in our analysis. For instance, levetiracetam has been established as the first-line antiepileptic drug for grade 2 brain tumors, but our study only considered the presence or absence of metastatic brain tumors. Further analysis could explore tumor grade, the type of seizure (focal or generalized), and the use of chemotherapy or antineoplastic drugs. Additionally, most studies in this meta-analysis did not statistically describe tumor progression, highlighting the need to investigate the impact of novel AEDs on tumor progression.

Our findings indicate that novel antiepileptic drugs are effective for seizure control in patients with brain tumors. Single-arm analysis revealed a treatment response rate of 72% and a seizure freedom rate of 34%. Sensitivity analysis demonstrated a significant difference in treatment response between monotherapy and combination therapy, suggesting that novel antiepileptic drugs are more effective as adjuvant therapy. Although more studies were conducted on lacosamide (LAC) and perampanel (PER), no significant difference in efficacy and adverse reactions between these two drugs in seizure control was observed. In contrast, clobazam exhibited the best efficacy. To validate our results, more randomized controlled trials are required.

## Author contributions

WZ: Writing – review & editing, Writing – original draft, Methodology, Conceptualization. QY: Writing – review & editing, Validation, Methodology. HW: Writing – review & editing, Supervision, Methodology, Conceptualization.

## References

[1] Miller KD Ostrom QT Kruchko C Patil N Tihan T Cioffi G. Brain and other central nervous system tumor statistics, 2021. CA Cancer J Clin. (2021) 71:381–406. doi: 10.3322/caac.21693, PMID: 34427324

[2] Fisher RS Acevedo C Arzimanoglou A Bogacz A Cross JH Elger CE. ILAE official report: a practical clinical definition of epilepsy. Epilepsia. (2014) 55:475–82. doi: 10.1111/epi.12550, PMID: 24730690

[3] Seidel S Wehner T Miller D Wellmer J Schlegel U Gronheit W. Brain tumor related epilepsy: pathophysiological approaches and rational management of antiseizure medication. Neurol Res Pract. (2022) 4:45–5. doi: 10.1186/s42466-022-00205-9, PMID: 36059029 PMC9442934

[4] Pallud J McKhann GM. Diffuse low-grade glioma-related epilepsy. Neurosurg Clin N Am. (2019) 30:43–54. doi: 10.1016/j.nec.2018.09.001, PMID: 30470404

[5] Kerkhof M Dielemans JC van Breemen MS Zwinkels H Walchenbach R Taphoorn MJ. Effect of valproic acid on seizure control and on survival in patients with glioblastoma multiforme. Neuro-Oncology. (2013) 15:961–7. doi: 10.1093/neuonc/not057, PMID: 23680820 PMC3688020

[6] Wirsching HG Morel C Gmur C Neidert MC Baumann CR Valavanis A. Predicting outcome of epilepsy after meningioma resection. Neuro-Oncology. (2016) 18:1002–10. doi: 10.1093/neuonc/nov303, PMID: 26683139 PMC4896539

[7] Slegers RJ Blumcke I. Low-grade developmental and epilepsy associated brain tumors: a critical update 2020. Acta Neuropathol Commun. (2020) 8:27. doi: 10.1186/s40478-020-00904-x, PMID: 32151273 PMC7063704

[8] Stritzelberger J Lang JD Mueller TM Reindl C Westermayer V Kostev K. Anti-seizure medication is not associated with an increased risk to develop cancer in epilepsy patients. J Neurol. (2021) 268:2185–91. doi: 10.1007/s00415-020-10379-4, PMID: 33484324 PMC8179889

[9] Slim K Nini E Forestier D Kwiatkowski F Panis Y Chipponi J. Methodological index for non-randomized studies (minors): development and validation of a new instrument. ANZ J Surg. (2003) 73:712–6. doi: 10.1046/j.1445-2197.2003.02748.x, PMID: 12956787

[10] Izumoto S Miyauchi M Tasaki T Okuda T Nakagawa N Nakano N. Seizures and tumor progression in glioma patients with uncontrollable epilepsy treated with perampanel. Anticancer Res. (2018) 38:4361–6. doi: 10.21873/anticanres.12737, PMID: 29970574

[11] Vecht C Duran-Pena A Houillier C Durand T Capelle L Huberfeld G. Seizure response to perampanel in drug-resistant epilepsy with gliomas: early observations. J Neuro-Oncol. (2017) 133:603–7. doi: 10.1007/s11060-017-2473-1, PMID: 28492978

[12] Saria MG Corle C Hu J Rudnick JD Phuphanich S Mrugala MM. Retrospective analysis of the tolerability and activity of lacosamide in patients with brain tumors: clinical article. J Neurosurg. (2013) 118:1183–7. doi: 10.3171/2013.1.JNS12397, PMID: 23451905

[13] Maschio M Zarabla A Maialetti A Fabi A Vidiri A Villani V. Quality of life, mood and seizure control in patients with brain tumor related epilepsy treated with lacosamide as add-on therapy: a prospective explorative study with a historical control group. Epilepsy Behav. (2017) 73:83–9. doi: 10.1016/j.yebeh.2017.05.031, PMID: 28623754

[14] Maschio M Pauletto G Zarabla A Maialetti A Ius T Villani V. Perampanel in patients with brain tumor-related epilepsy in real-life clinical practice: a retrospective analysis. Int J Neurosci. (2019) 129:593–7. doi: 10.1080/00207454.2018.1555160, PMID: 30507318

[15] Maschio M Zarabla A Maialetti A Giannarelli D Koudriavtseva T Villani V. Perampanel in brain tumor-related epilepsy: observational pilot study. Brain Behav. (2020) 10:e01612. doi: 10.1002/brb3.1612, PMID: 32285623 PMC7303381

[16] Heugenhauser J Iglseder S Muigg A Kerschbaumer J Stockhammer G Nowosielski M. Perampanel in brain tumor and SMART-syndrome related epilepsy—a single institutional experience. J Neurol Sci. (2021) 423:117386–6. doi: 10.1016/j.jns.2021.117386, PMID: 33706200

[17] Coppola A Zarabla A Maialetti A Villani V Koudriavtseva T Russo E. Perampanel confirms to be effective and well-tolerated as an add-on treatment in patients with brain tumor-related epilepsy (PERADET study). Front Neurol. (2020) 11:592. doi: 10.3389/fneur.2020.00592, PMID: 32695064 PMC7336340

[18] Toledo M Molins A Quintana M Santamarina E Martinez-Ricarte F Martinez-Saez E. Outcome of cancer-related seizures in patients treated with lacosamide. Acta Neurol Scand. (2018) 137:67–75. doi: 10.1111/ane.12809, PMID: 28832891

[19] Villanueva V Saiz-Diaz R Toledo M Piera A Mauri JA Rodriguez-Uranga JJ. NEOPLASM study: real-life use of lacosamide in patients with brain tumor-related epilepsy. Epilepsy Behav. (2016) 65:25–32. doi: 10.1016/j.yebeh.2016.09.033, PMID: 27863278

[20] Ruda R Pellerino A Franchino F Bertolotti C Bruno F Mo F. Lacosamide in patients with gliomas and uncontrolled seizures: results from an observational study. J Neuro-Oncol. (2018) 136:105–14. doi: 10.1007/s11060-017-2628-0, PMID: 29030718

[21] Mo F Meletti S Belcastro V Quadri S Napolitano M Bello L. Lacosamide in monotherapy in BTRE (brain tumor-related epilepsy): results from an Italian multicenter retrospective study. J Neuro-Oncol. (2022) 157:551–9. doi: 10.1007/s11060-022-03998-6, PMID: 35397759

[22] Maschio M Dinapoli L Mingoia M Sperati F Pace A Pompili A. Lacosamide as add-on in brain tumor-related epilepsy: preliminary report on efficacy and tolerability. J Neurol. (2011) 258:2100–4. doi: 10.1007/s00415-011-6132-8, PMID: 21674196

[23] Zoccarato M Basile AM Padovan M Caccese M Zagonel V Lombardi G. Eslicarbazepine in patients with brain tumor-related epilepsy: a single-center experience. Int J Neurosci. (2021) 131:879–84. doi: 10.1080/00207454.2020.1759590, PMID: 32316814

[24] Brahmbhatt N Stupp R Bushara O Bachman E Schuele SU Templer JW. Efficacy of clobazam as add-on therapy in brain tumor-related epilepsy. J Neuro-Oncol. (2021) 151:287–93. doi: 10.1007/s11060-020-03664-9, PMID: 33398534

[25] Sepulveda-Sanchez JM Conde-Moreno A Baron M Pardo J Reynes G Belenguer A. Efficacy and tolerability of lacosamide for secondary epileptic seizures in patients with brain tumor: a multicenter, observational retrospective study. Oncol Lett. (2017) 13:4093–100. doi: 10.3892/ol.2017.5988, PMID: 28599411 PMC5452997

[26] Ruda R Houillier C Maschio M Reijneveld JC Hellot S De Backer M. Effectiveness and tolerability of lacosamide as add-on therapy in patients with brain tumor-related epilepsy: results from a prospective, noninterventional study in European clinical practice (VIBES). Epilepsia. (2020) 61:647–56. doi: 10.1111/epi.16486, PMID: 32329527 PMC7384112

[27] Maschio M Maialetti A Mocellini C Domina E Pauletto G Costa C. Effect of brivaracetam on efficacy and tolerability in patients with brain tumor-related epilepsy: a retrospective multicenter study. Front Neurol. (2020) 11:813. doi: 10.3389/fneur.2020.00813, PMID: 32973649 PMC7466736

[28] Fairclough S Goodden J Chumas P Mathew R Maguire M. Levetiracetam as a first-line antiseizure medication in WHO grade 2 glioma: time to seizure freedom and rates of treatment failure. Epilepsia. (2023) 64:857–65. doi: 10.1111/epi.17508, PMID: 36636895

[29] Belcastro V Pisani LR Bellocchi S Casiraghi P Gorgone G Mula M. Brain tumor location influences the onset of acute psychiatric adverse events of levetiracetam therapy: an observational study. J Neurol. (2017) 264:921–7. doi: 10.1007/s00415-017-8463-6, PMID: 28315958

[30] Van Der Meer PB Dirven L Fiocco M Vos MJ Kouwenhoven MCM Van Den Bent MJ. Effectiveness of antiseizure medication duotherapies in patients with glioma: a multicenter observational cohort study. Neurology. (2022) 99:E999–E1008. doi: 10.1212/WNL.0000000000200807, PMID: 36219797 PMC9519253

[31] Maschio M Aguglia U Avanzini G Banfi P Buttinelli C Capovilla G. Management of epilepsy in brain tumors. Neurol Sci. (2019) 40:2217–34. doi: 10.1007/s10072-019-04025-9 31392641

[32] Fan Z Sehm T Rauh M Buchfelder M Eyupoglu IY Savaskan NE. Dexamethasone alleviates tumor-associated brain damage and angiogenesis. PLoS One. (2014) 9:e93264. doi: 10.1371/journal.pone.0093264, PMID: 24714627 PMC3979667

[33] Stewart-Amidei C. Managing symptoms and side effects during brain tumor illness. Expert Rev Neurother. (2005) 5:S71–6. doi: 10.1586/14737175.5.6.S71 16274273

[34] Koehler PJ. Use of corticosteroids in neuro-oncology. Anti-Cancer Drugs. (1995) 6:19–33. doi: 10.1097/00001813-199502000-00002 7756680

[35] Usery JB Michael LM 2nd Sills AK Finch CK. A prospective evaluation and literature review of levetiracetam use in patients with brain tumors and seizures. J Neuro-Oncol. (2010) 99:251–60. doi: 10.1007/s11060-010-0126-8, PMID: 20146087

[36] Lange F WeSslau K Porath K Hornschemeyer J Bergner C Krause BJ. AMPA receptor antagonist perampanel affects glioblastoma cell growth and glutamate release *in vitro*. PLoS One. (2019) 14:–e0211644. doi: 10.1371/journal.pone.0211644, PMID: 30716120 PMC6361447

[37] Rizzo A Donzelli S Girgenti V Sacconi A Vasco C Salmaggi A. *In vitro* antineoplastic effects of brivaracetam and lacosamide on human glioma cells. J Exp Clin Cancer Res. (2017) 36:76. doi: 10.1186/s13046-017-0546-9, PMID: 28587680 PMC5460451

[38] Kwok CS Johnson EL Krauss GL. Comparing safety and efficacy of “third-generation” antiepileptic drugs: long-term extension and post-marketing treatment. CNS Drugs. (2017) 31:959–74. doi: 10.1007/s40263-017-0480-6, PMID: 29204953

